# Functionalization of polyacrylamide for nanotrapping positively charged biomolecules†

**DOI:** 10.1039/c8ra07764a

**Published:** 2019-05-16

**Authors:** Nadejda Davydova, Xavier R. Rodriguez, Carlos Blázquez, Andrés Gómez, Igor Perevyazko, Judith Guasch, Vladimir Sergeev, Elena Laukhina, Imma Ratera, Jaume Veciana

**Affiliations:** A. N. Nesmeyanov Institute of Organoelement Compounds of Russian Academy of Sciences Vavilova St. 28 119991 Moscow Russia; CIBER de Bioingeniería, Biomateriales y Nanomedicina (CIBER-BBN) Campus UAB 08193 Barcelona Spain; Department of Molecular Nanoscience and Organic Materials, Institute of Materials Science of Barcelona (ICMAB-CSIC) Campus UAB 08193 Barcelona Spain laukhina@icmab.es; Dynamic Biomimetics for Cancer Immunotherapy, Max Planck Partner Group, ICMAB-CSIC Campus UAB 08193 Barcelona Spain jguasch@icmab.es; SPM Service, Institute of Materials Science of Barcelona (ICMAB-CSIC) Campus UAB 08193 Barcelona Spain a.gomez@icmab.es; Department of Molecular Biophysics and Polymer Physics, St. Petersburg State University Ulyanovskaya St. 1 198504 St. Petersburg Russia

## Abstract

Engineering new materials which are capable of trapping biomolecules in nanoscale quantities, is crucial in order to achieve earlier diagnostics in different diseases. This article demonstrates that using free radical copolymerization, polyacrylamide can be successfully functionalized with specific synthons for nanotrapping positively charged molecules, such as numerous proteins, through electrostatic interactions due to their negative charge. Specifically, two functional random copolymers, acrylamide/acrylic acid (1) and acrylamide/acrylic acid/*N*-(pyridin-4-yl-methyl)acrylamide (2), whose negative net charges differ in their water solutions, were synthetized and their ability to trap positively charged proteins was studied using myoglobin as a proof-of-concept example. In aqueous solutions, copolymer 1, whose net charge for a 100 chain fragment (*Q*_pH 6_/*M*) is −1.323 × 10^−3^, interacted with myoglobin forming a stable monodisperse nanosuspension. In contrast, copolymer 2, whose value of *Q*_pH 6_/*M* equals −0.361 × 10^−3^, was not able to form stable particles with myoglobin. Nevertheless, thin films of both copolymers were grown using a dewetting process, which exhibited nanoscale cavities capable of trapping different amounts of myoglobin, as demonstrated by bimodal AFM imaging. The simple procedures used to build protein traps make this engineering approach promising for the development of new materials for biomedical applications where trapping biomolecules is required.

## Abstract 2

Engineering new materials which are capable of trapping biomolecules in nanoscale quantities, is crucial in order to achieve earlier diagnostics in different diseases.

## Graphical abstract

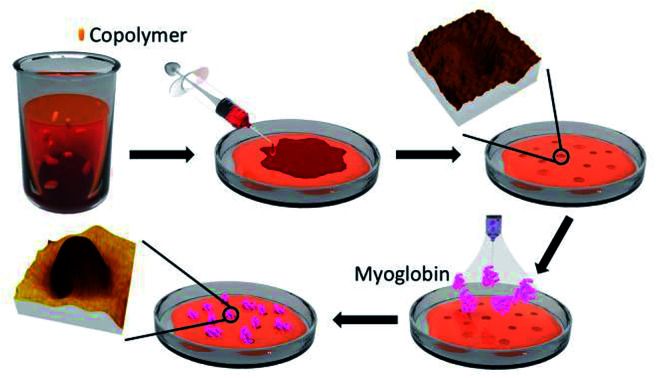

## Introduction

The manipulation of biomolecules at the nanoscale has a great potential in biomedical applications,^1–11^*e.g.* in achieving early diagnostics for different diseases. Thus, there is an increasing need to develop novel materials and methods to trap small amounts of biomolecules. Currently, the most widely used nanomanipulation methods are based on optical properties, such as the optical tweezers.^12–15^ However, optical tweezers can only trap sufficiently polarizable small objects, thus they are not effective for weakly polarized macromolecules. To overcome this limitation, alternative trapping approaches based on electrokinetic,^16^ near field photonics,^17^ and acoustic methods^18^ have been developed. Recently, an electrostatic method that uses topological modulations of the gap between two fluidic slit surfaces that acquire a net charge on exposure to water was proposed.^19^ Despite the fact that several nanomanipulation methods have been successfully developed, stable trapping of nanometer-sized objects remains challenging. To overcome this problem, the design and synthesis of novel bioactive polymers emerges as a promising approach, given that their functional groups and therefore their interactions with biomolecules and nanoparticles can be easily tailored thanks to the great versatility of synthetic chemistry. Taking into account that the basis of different sensors and diagnostic systems are specific chemical interactions,^20–22^ we developed new copolymers capable of establishing electrostatic and π–π interactions with DNA.^23–25^ It was demonstrated that controlled free radical copolymerization enables the preparation of water soluble bioactive cationic acrylamide-based copolymers with desired amounts of different monomeric units.^23,25^ Moreover, such copolymers were able to form thin films with arrays of submicro- and nanoscale cavities that successfully trapped DNA at the nanoscale.^24^

**Fig. 1:**
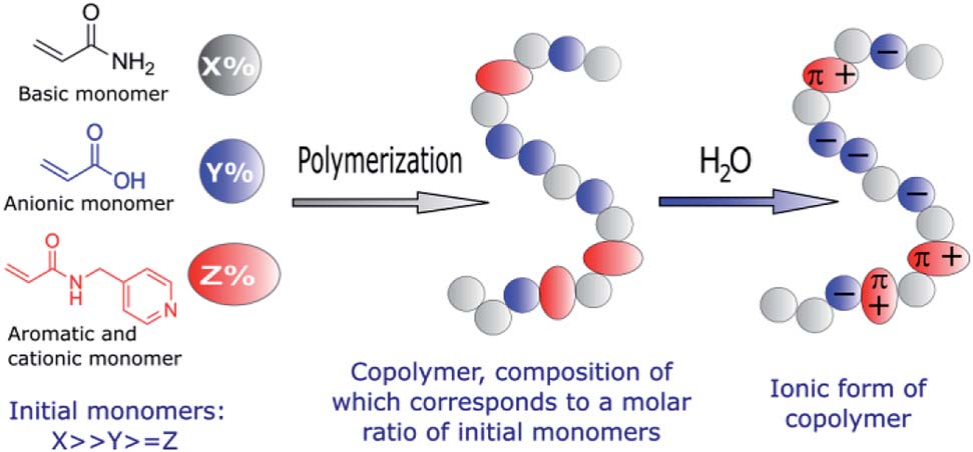
Schematic representation of the functional groups used to prepare amphoteric copolymers with desired net negative charges in aqueous solutions through free-radical copolymerization.

Encouraged by these results, we explored the preparation of anionic acrylamide-based copolymers to trap biomolecules, which are positively charged in aqueous solutions, such as numerous proteins. With this objective, an analogous synthetic procedure was employed to prepare anionic acrylamide-based copolymers with functional groups that are negatively charged in aqueous solutions (Fig. 1). Additionally, functional groups capable of forming π–π interactions with different biomolecules were added to one of the acrylamide-based copolymers.

**Fig. 2:**
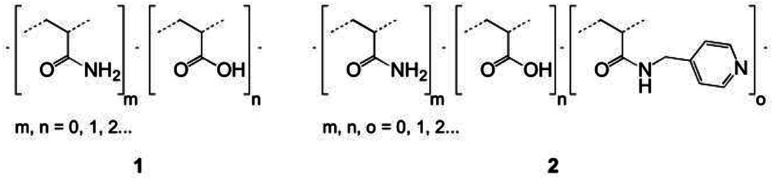
Skeletal formulas of random copolymers 1 and 2.

Specifically, two copolymers with predetermined molar ratios of their monomeric units consisting of acrylamide (90%)/acrylic acid (10%) (1) and acrylamide (91%)/acrylic acid (5%)/*N*-(pyridin-4-yl-methyl)-acrylamide (4%) (2) were synthesized (Fig. 2).

To use such anionic copolymers as nanotrapping components in devices, both copolymers were deposited by a simple dewetting process on highly flexible piezoresistive organic conductive supports,^26^ potentially capable of electrically detecting biomolecules such as proteins.^21,22^ The copolymers self-assembled into thin films containing nanoscale cavities of different sizes, which were able to successfully trap the positively charged protein myoglobin, mainly through electrostatic interactions. This protein was selected to prove the trapping model due to its use as a sensitive marker for muscle injury, which is considered a potential marker for heart attack.^27–29^

## Experimental

### Synthesis of the copolymers: acrylamide/acrylic acid (1) and acrylamide/acrylic acid/*N*-(pyridin-4-yl-methyl)acrylamide (2)

#### Synthesis of acrylamide/acrylic acid copolymer (1)

24 μl (0.16 mmol) of *N*,*N*,*N*′,*N*′-tetramethylethylenediamine (TEMED) were added to a solution of 0.639 g (0.009 mol) of acrylamide and 0.094 g (0.001 mol) of sodium acrylate in 20 ml of water. The reaction mixture was sparged with argon for 20 min and heated to 50 °C. A solution of 8 mg (0.035 mmol) of ammonium persulfate (APS) in 0.5 ml water was added to the reaction mixture. The polymerization was conducted under stirring for 1 h at 50 °C. The reaction mixture was cooled down to room temperature, dialyzed against low molecular weight (up to 10 kDa) fractions, and freeze-dried. 0.571 g of copolymer 1 were obtained, resulting in a 75% yield. According to the acid–base titration of the carboxylic groups, the molar ratio of acrylamide/acrylic acid was 0.90 : 0.10.

IR (*ν*, cm^−1^, KBr): 3407 (OH), 3200 (NH_2_), 2928 (CH), 1661 (C=O), 1617 (NH_2_), 1454, 1413 (CH, C–C).

#### Synthesis of acrylamide/acrylic acid/*N*-(pyridin-4-yl-methyl)acrylamide copolymer (2)

24 μl (0.16 mmol) of TEMED were added to a solution of 0.468 g (0.0066 mol) of acrylamide, 0.033 g (0.00035 mol) of sodium acrylate and 0.056 g (0.00035 mol) of *N*-(pyridin-4-yl-methyl)acrylamide in 20 ml of water. The reaction mixture was sparged with argon for 20 min and heated to 50 °C. A solution of 8 mg (0.035 mmol) of APS in 0.5 ml water was added to the reaction mixture. The polymerization and work-up procedure was identical to that of copolymer 1. Thus, 0.361 g of copolymer 2 were obtained, resulting in a 64% yield. The acid–base titration and integral intensities of the ^1^H-NMR signals corresponding to the protons of CH and NH groups determined the molar ratio of the components in copolymer 2 as acrylamide (0.91)/acrylic acid (0.05)/*N*-(pyridin-4-yl-methyl)-acrylamide (0.04).

IR (*ν*, cm^−1^, KBr): 3414 (OH), 3200 (NH), 2925 (CH), 1663 (C=O), 1619 (NH_2_), 1451, 1415 (CH, C–C).

### Materials and methods

Myoglobin from equine heart (purity ≥ 90%) and the negatively charged circular DNA (pBR322) were purchased from Sigma Aldrich and used without further treatment. The molar mass of the myoglobin was taken from the literature.^30^

^1^H-NMR spectra were recorded in D_2_O using a Bruker Model ARX (300 MHz) spectrometer, whereas IR spectra were studied in the range of 3500–1000 cm^−1^ using a Bruker Tensor 37. Particle size measurements in solution were performed by dynamic light scattering (DLS) using a Zetasizer Nano ZS device and the Zetasizer Software version 7.11 to analyze the data. Scanning electron microscopy (SEM) images were obtained in a microscope QUANTA FEI 200 FEG-ESEM.

The AFM characterization was performed using a Keysight 5500LS AFM with a signal access box option. The equipment was configured to perform bimodal imaging by feeding the Lock-in Drive 2 towards a summing amplifier, while the output of the amplifier was connected to the piezo ditter present in the scanner head. All the images were performed with the same type of probe, an AppNano FORT tip, in dry conditions. For the dynamic mode, the tip was excited at the first resonance mode with standard parameters,^31^ to perform the scans. For the bimodal images, we excited the first and second resonance modes of the cantilever, ∼67 and ∼425 kHz, respectively. The setpoint of the instrument was set to ∼15 nm, while the second resonance mode was set to ∼10% of the first resonance mode amplitude value. Depending on the specific conditions of the sample, the parameters were varied by 30% maximum. By using a small second resonance amplitude and substantial higher setpoint values, the measurements were not affected by possible electrostatic artifacts presented in bimodal AFM imaging.^32,33^

### Molar mass determination

The molar masses of the copolymers were evaluated by the sedimentation-diffusion analysis in the analytical ultracentrifuge using the modified Svedberg equation:I
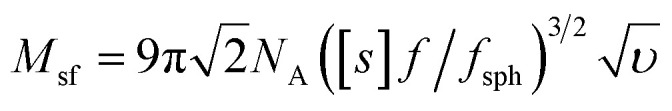
where *N*_A_ is the Avogadro's number, 
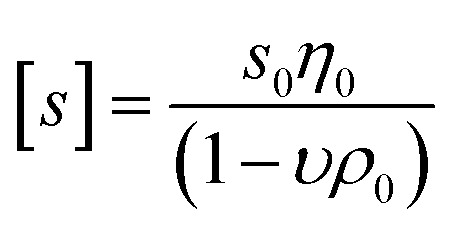
 is the intrinsic sedimentation coefficient, *f*/*f*_sph_ is the frictional ratio, and *υ* is the partial specific volume. The partial specific volume was determined by the classical approach *via* density measurements of the copolymer solutions at different concentrations and constitutes *υ* = 0.728 cm^3^ g^−1^ (Fig. S1†).

### Estimation of the net charge (*Q*) of copolymers in aqueous solutions at pH = 6

The charge was estimated using the Henderson–Hasselbalch equation (II):^34^

II *Q*(pH) = *aq*_1_ + *bq*_2_

where *a* and *b* are the amount of functional groups based on acrylic acid and *N*-(pyridin-4-yl-methyl)acrylamide, respectively; *q*_1_ and *q*_2_ are the charges of these functional groups in phosphate buffered saline (PBS; pH = 6). The values of *q*_1_ and *q*_2_ were calculated using the following formulas named (III) and (IV):^34^

III *q*_1_ = (−1)/[1 + 10^(p*K*_a_−pH)^]

IV *q*_2_ = 1/[1 + 10^(pH−p*K*_b_)^]

where p*K*_a_ and p*K*_b_ are the dissociation constants of the functional groups of copolymers 1 and 2 (Table S1†). Additionally, the table presents the p*K*_a_ values of myoglobin used for the estimation of its net charge at pH = 6.

### Complexation behavior of copolymers 1 and 2 with myoglobin in aqueous solutions

The interaction of copolymers 1 and 2 with myoglobin in aqueous solutions was analyzed by DLS. The copolymers were suspended in Milli-Q® water at a concentration of 1 mg ml^−1^ at room temperature. Myoglobin was diluted at a concentration of 1 mg ml^−1^ in PBS (pH = 6) also at room temperature. To study the interactions of copolymers with myoglobin, 245 μl of the buffer solution of myoglobin were added into a 1 ml suspension of either copolymer 1 or 2 (Table 2). 1 ml of the resulting suspension was measured at least 3 times with 3 single runs per measurement at 25 °C.

**Table 1:** Molar masses and net charge (*Q*) of myoglobin and copolymers at pH = 6

Compound	*M* [g mol^−1^]	*Q*_pH 6_ a	*Q*_pH 6_/*M*
Myoglobin	16 951	7.047	0.416 × 10^−3^
1	115 100 (7.122^b^)	−152.22 (−9.419^b^)	−1.323 × 10^−3^
2	76 300 (7.386^b^)	−27.51 (−2.663^b^)	−0.361 × 10^−3^

a *Q*_pH 6_ represents the overall electric charge as a sum of elementary charges (elementary charge = 1.6 × 10^−19^ C).

b Molecular mass and charge of a 100 chain fragment.

**Table 2:** Sample preparation and DLS data collected at 25 °C

Compound A (solvent)^a^	Compound B^b^ (solvent)^a^	Mass (molarity) in a 1 ml cuvette	DLS data	
			Diameter, [nm]	PDI
1 (1000 μl water)	# (245 μl PBS)	1: 803 μg (0.07 × 10^−3^ μM)	17 ± 10	0.674
			40 ± 20	
			600 ± 300	
2 (1000 μl water)	# (245 μl PBS)	2: 803 μg (0.1 × 10^−3^ μM)	30 ± 12	0.408
			430 ± 173	
Myoglobin (1000 μl PBS)	# (#)	1000 μg	5 ± 2	0.201
1 (1000 μl water)	Myoglobin (245 μl PBS)	1: 803 μg, myoglobin: 197 μg	190 ± 70	0.197
2 (1000 μl water)	Myoglobin (245 μl PBS)	2: 803 μg, myoglobin: 197 μg	8 ± 2	0.786
			30 ± 10	
			430 ± 173	

a All PBS used was equilibrated to have a pH = 6.

b #: no chemical.

### Preparation of a flexible highly piezoresistive bilayer support: polycarbonate/00*l* oriented polycrystalline layer of α-(BEDT-TTF)_2_I_3_

A 20–25 μm thick polycarbonate/00*l* oriented α-(BEDT-TTF)_2_I_3_ bilayer film was prepared as described previously.^26^ The film was fabricated at the bottom of a glass Petri dish in a climate chamber “Memmert HPP108” operated at 20 °C and relative humidity of 40% (Fig. S2†). The formation of the *00l* oriented layer of α-(BEDT-TTF)_2_I_3_ was confirmed by the X-ray diffraction pattern that demonstrated a set of reflections typical for 001 oriented α-(BEDT-TTF)_2_I_3_ crystallites (Fig. S3†) and SEM (Fig. S4†).

### Preparation of thin nanostructured layers of copolymers 1 and 2 and their interaction with myoglobin

Using a dewetting process, thin films (~10 nm) of both copolymers were fabricated over the conducting supports. Water solutions of copolymers 1 and 2 with a concentration of 1 mg ml^−1^ were prepared at ambient conditions and deposited by the drop-casting technique (4 μl divided in two drops). Films were then kept in a climate chamber “Memmert HPP108” at 20 °C and a relative humidity of 40% for 3 h. Then, a 2 μl solution of myoglobin (0.1 mg ml^−1^) in PBS was deposited over each film. Both the presence of nanocavities and their filling was confirmed by bimodal AFM.

## Results and discussion

**Fig. 3:**
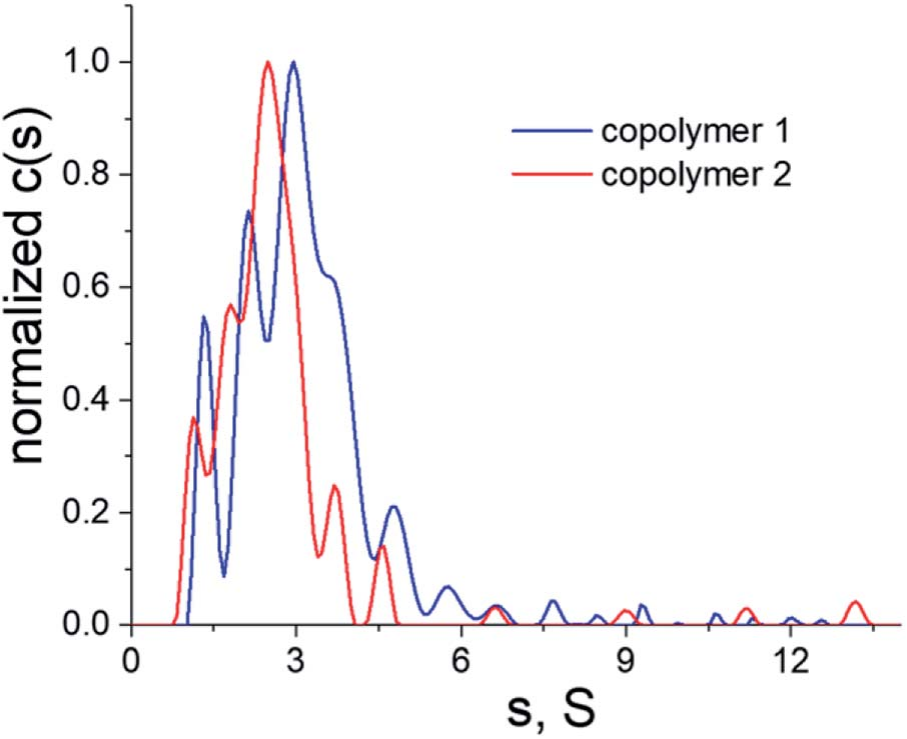
Differential distributions of sedimentation coefficients of copolymers 1 (blue line) and 2 (red line) in a 0.2 M NaCl solution in water, *T* = 25 °C.

Given that the electrostatic interactions between the anionic copolymers and the cationic myoglobin were expected to be responsible for the nanotrapping, the influence of the net charge of the copolymers on the protein complexation was studied. Moreover, the effect of the net charge and physicochemical characteristics of the copolymers on the morphology of the thin films was also analyzed. To this end, copolymers 1 and 2 (Fig. 2), with different net charges in aqueous solutions, were synthesized, and their complexation behavior with myoglobin at pH = 6 was examined. Nevertheless, to avoid electrostatic interactions in solution and to determine the correct molecular characteristics of the copolymers, all hydrodynamic studies were performed in a 0.2 M NaCl solution in water. Fig. 3 shows a comparison of the distributions of the sedimentation coefficients for copolymers 1 and 2. Both polymeric systems were found to have relatively high dispersity in terms of broad distributions of sedimentation coefficients. Besides the sedimentation coefficients, modern analytical procedures allow to extract information on the related diffusion coefficients, which are represented in terms of frictional ratios – *f*/*f*_sph_, where *f* is the translation friction coefficient of a studied particle/macromolecule and *f*_sph_ is that of an equivalent sphere having the same mass and density. Both, the sedimentation coefficients and frictional ratios were obtained at different copolymer concentrations and extrapolated to zero concentration (Fig. S5 and S6†). Based on the determined values of frictional ratio, the molar mass was evaluated using the modified Svedberg relationship (I). The calculated values were 115 100 g mol^−1^ and 76 300 g mol^−1^ for the copolymers 1 and 2, respectively (Table 3).

**Table 3:** Hydrodynamic characteristics and molar mass of copolymers 1 and 2 in a 0.2 M NaCl solution in water, *T* = 25 °C

Compound	*s*_o_ [S]	(*f*/*f*_sph_)_0_	*D*_0_ × 10^7^ [cm^2^ s^−1^]	*M*_sf_ [g mol^−1^]
1	3.65	2.7	2.8	115 100
2	2.90	2.5	3.5	76 300

Given that both copolymers 1 and 2 have similar characteristics and consist of a 90% of repeated units of acrylamide, their physical properties (*e.g.* viscosity, solubility, mechanical strength, *etc.*) are expected to be very similar. In contrast, the functionalization of polyacrylamide with different specific groups (<10%), which readily become charged in water, result in substantial differences in terms of the negative net charge between copolymers. Indeed, the estimation of the net charge of both copolymers (*Q*) (Table 1) showed that the negative net charge of copolymer 2 is approximately six times smaller than that of copolymer 1. Moreover, the small positive charge resulting from the system copolymer 2/myoglobin in aqueous solutions could make the formation of stable complexes difficult.

**Fig. 4:**
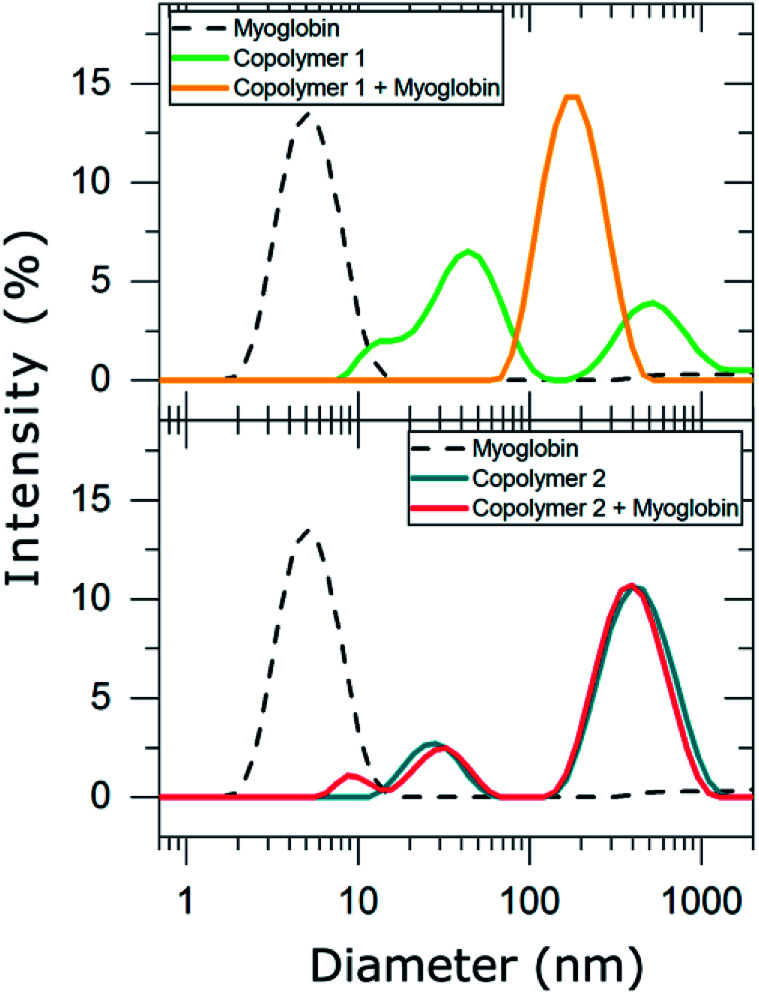
DLS graphs showing the change in size distribution of the polymeric particles after adding myoglobin. Myoglobin (black dotted line), copolymer 1 (green line), copolymer 1 with myoglobin (yellow line), copolymer 2 (blue line), and copolymer 2 with myoglobin (red line).

Consequently, DLS experiments were performed to experimentally evaluate the complexation behavior of copolymers 1 and 2 with myoglobin (Table 2 and Fig. 4).

In PBS diluted solutions, compounds 1 and 2 formed disperse nanosuspensions with a polydispersity index (PDI) of 0.674 and 0.408, respectively. These high values of PDI are indicative of unstable distributions of the polymeric particles formed in such dispersion medium. In contrast, myoglobin dispersed in PBS exhibited a long term stable unimodal particle distribution with a hydrodynamic particle diameter of 5 ± 2 nm and a PDI of 0.201. As myoglobin is described to be 5.7 nm long in one axis and 3.1 nm in the other according to crystallographic data,^35^ we suggest that dispersing myoglobin in PBS resulted in a monomolecular suspension. The addition of myoglobin to copolymer 1 led to the formation of a new population of nanoparticles whose hydrodynamic diameter was 190 ± 72 nm (PDI = 0.20). This result evidences that copolymer 1 forms stable complexes with myoglobin and may be considered a good candidate for trapping myoglobin in solution. In contrast, copolymer 2 was not able to complex myoglobin, *i.e.* the mixture of the copolymer and protein resulted in a very unstable particle distribution (PDI = 0.786), which mainly corresponds to the particle distribution of copolymer 2 and small particles (8 ± 2 nm) compatible with myoglobin dimers. Thus, only suspensions that contained the highly charged copolymer 1 resulted in interactions between the synthesized anionic copolymers and the cationic protein myoglobin. It is also worth mentioning that other parameters, *i.e.* the charge density of the copolymers might also influence the complex formation, *e.g.* the aromatic units of copolymer 2 might result in a 3D organization of the copolymer with the negative charges less available to myoglobin in comparison with copolymer 1.

**Fig. 5:**
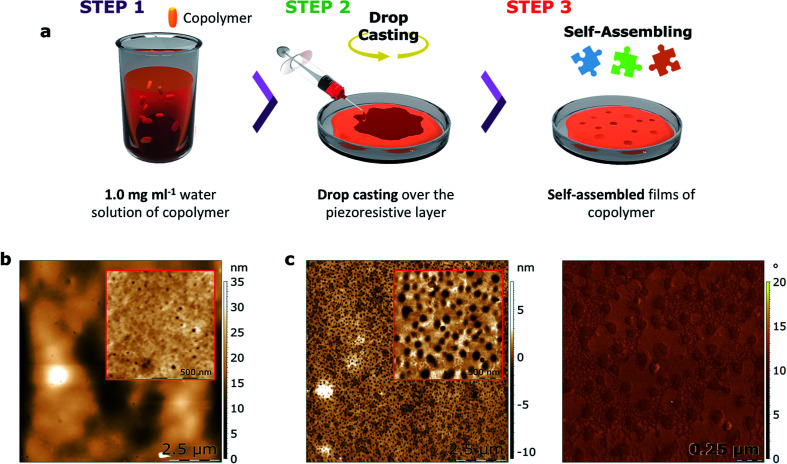
Formation of nanocavities. (a) Schematic representation of the preparation of copolymer-based thin films, (b) AFM topography image and zoom-in scan performed in bimodal phase of a self-assembled film based on copolymer 1, indicating the formation of a large number of nanocavities with an average diameter of 17 nm, and (c) AFM topography image and zoom-in scan of a self-assembled film based on copolymer 2 performed in bimodal phase (left) and bimodal AFM phase image obtained simultaneously with the inset of (c) (right); the AFM data confirm the formation of nanocavities whose average diameter is 108 nm.

With the objective of studying the capacity of such copolymers to be used in a device for protein nanotrapping, thin films of both copolymers 1 and 2 were prepared by drop-casting of their water solutions and self-assembly over the piezoresistive α-(BEDT-TTF)_2_I_3_-based support in ambient conditions after a dewetting process. As shown by AFM (Fig. 5 and S7†), nanocavities formed at the surfaces of the films of both copolymers. To discard the formation of nanoholes, bimodal phase images were performed, which are sensitive to changes in the mechanical properties of a sample. As determined by a sample of a block copolymer with known morphology and mechanical properties, lower phases represent stiff materials, while higher phases represent soft materials (Fig. S9†). The absence of contrast in the phase image indicates the formation of cavities as opposed to holes, as the presence of holes would expose the piezoresistive crystalline layer, which is a harder material than the copolymers.

The morphology of the cavities formed on the self-assembled films of copolymers 1 and 2 show significant differences in mean depth (*H*). Specifically, the cavities in the film formed by copolymer 1 are about three times smaller compared with the cavities formed by copolymer 2 (Table 4). Moreover, the smaller diameter (*D*) of the copolymer 1-based cavities results in a surface area (*S*) occupied by these cavities that is about thirty times smaller than the surface area of copolymer 2. Taking into account that both films were self-assembled using the same conditions, it is suggested that the net charge of the copolymers significantly effects the subtle interplay between the differing contributions to the free energy of the α-(BEDT-TTF)_2_I_3_/copolymer/air multilayered system. Nevertheless, the influence of steric factors on the film formation related to the aromatic units of copolymer 2 cannot be fully ruled out.

**Table 4:** Morphology AFM data of the nanocavities formed on the films of copolymers 1 and 2, where “*H*” stands for mean depth, “*A*” is the area of the cavities, “*S*” is the surface area occupied by the cavities, “*D*” is the mean diameter, and “*V*_c_” is the mean volume of the cavities

Compound	*H* [nm]	*A* [nm^2^]	*S* [%]	*D* [nm]	*V*_c_ [nm^3^]
1	3.2	207	0.7	17	1161
2	8.7	4130	20	108	62 800

**Fig. 6:**
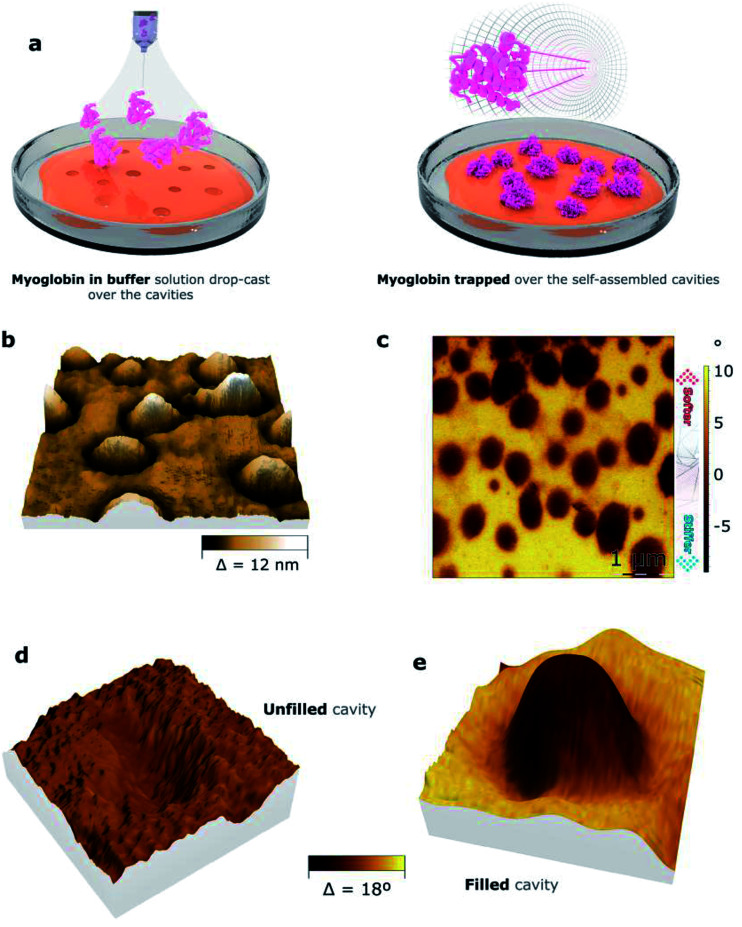
Protein nanotrapping. (a) Scheme of myoglobin deposition onto the self-assembled copolymeric films, (b) 3D representation of an AFM topography image of the myoglobin-filled self-assembled copolymer 2 film, (c) bimodal AFM phase image of the myoglobin-filled self-assembled copolymer 2 film, 3D representations of (d) an unfilled and (e) a filled cavity of the copolymer 2 film, in which colors represent the mechanical properties obtained through bimodal AFM phase imaging, while the roughness represents the topographic information.

To analyze the ability of the nanocavities formed by copolymers 1 and 2 to trap positively charged biomolecules, a solution of myoglobin in PBS was applied over the films by drop-casting. The resulting surfaces were studied by bimodal AFM imaging, which acquires both mechanical and topographic properties simultaneously (Fig. 6, S8,† and Table 5). The 3D representation of the topography images of both films demonstrate the formation of protrusions, suggesting the filling of the cavities by myoglobin. Indeed, the projected area of the protrusions, which were *ca.* 1% for copolymer 1 and *ca.* 13% for copolymer 2 (“*S*”, Table 5), nearly matches the surface area occupied by the cavities for both copolymers, which were *ca.* 1% and *ca.* 20%, respectively (“*S*”, Table 4). To assure that the protrusions corresponded to myoglobin, the mechanical properties of the films were assessed (Fig. 6c). Indeed, bimodal AFM images proved that the mechanical properties of the protrusions differ from the ones of the rest of the film. Moreover, the elastic modulus of the protrusions was estimated to be ten times higher than the polymer background (Fig. S9†). This result suggests that the protrusions might be crystalline-like aggregates, which pre-fitted the cavities, while the copolymeric material at the rest of the surface area remained intact.

**Table 5:** Morphology AFM data of the filled nanocavities formed on the films of copolymers 1 and 2, where “*h*” is the mean height of the protrusions, “*A*_filled_” is the area of filled cavities, “*S*” is the surface area occupied by the protrusions, and “*V*_p_” is the average volume of the protrusions

Compound	*h* [nm]	*A*_filled_ [nm^2^]	*S* [%]	*V*_p_ [nm^3^]
1	7.8	255	1.3	1104
2	5.8	3410	13.4	35 600

To explain this data, we propose the following mechanism. Initially, the surface of the copolymer-based films become swollen and negatively charged due to the addition of the buffer solution of myoglobin. The swollen cavities, which are probably able to adjust to the geometry of the myoglobin-based particles, provide a larger number of Coulomb interactions in comparison with the flat surface areas. Thus, myoglobin gets trapped by the cavities and under the dewetting process, myoglobin protrusions appear (Fig. S10†). The copolymer-based nanocavities could be therefore considered nucleation centers for myoglobin-containing salts.^35^

Finally, to corroborate the key role of Coulomb interactions, water solutions of either myoglobin or negatively charged circular DNA (pBR322) were prepared and added over thin films of copolymer 2 (Fig. S11†). As shown by AFM, the surface topography obtained depended on the type of added biomolecule. In the case of DNA, the cavities disappeared and some DNA-based nanostructures were randomly deposited over the surface. On the other hand, adding a water solution of myoglobin (pH ≈ 7), in which the protein was significantly less charged than in PBS solutions, resulted in the deposition of myoglobin-based structures apart from the cavities.

## Conclusions

We show that a simple free radical copolymerization process permits to synthesize anionic polyacrylamide-based copolymers with different synthons in precise ratios, whose negative net charges significantly differ in their aqueous solutions. DLS studies revealed that copolymer 1 is able to trap positively charge biomolecules such as myoglobin in solution forming a stable monodisperse nanosuspension, whereas copolymer 2 does not form stable complexes with the protein. However, bimodal AFM confirmed that both copolymers self-assembled as thin films containing nanoscale cavities, which showed the ability to trap the positively charged myoglobin. Thus, the proposed approach to engineer novel functional organic polymeric materials for molecular trapping has been validated, which could be further developed to engineer biosensors.

## Author contributions

The manuscript was written through contributions of all authors.

## Conflicts of interest

There are no conflicts to declare.

## Supplementary Material

RA-009-C8RA07764A-s001
