# Supplementary material for: Functionalization of polyacrylamide for nanotrapping positively charged biomolecules
Source: RSC Adv. 2019 May 16;9(27):15402–9. doi: 10.1039/c8ra07764a (PMC9064249; doi:10.1039/c8ra07764a)
Supplement: RA-009-C8RA07764A-s001 [file RA-009-C8RA07764A-s001.pdf]

Electronic Supplementary Material (ESI) for RSC Advances.
This journal is © The Royal Society of Chemistry 2019

Supplementary Information

Functionalization of polyacrylamide for nanotrapping positively charged biomolecules

Nadejda Davydova,[1,‡] Xavier R. Rodriguez,[2,3,4,‡] Carlos Blázquez,[2,3] Andrés Gómez,[5,*] Igor Perevyazko,[6] Judith Guasch,[2,3,4,*] Vladimir Sergeev,[1] Elena Laukhina,[2,3,*] Imma Ratera,[2,3] and Jaume Veciana[2,3]

[1] A. N. Nesmeyanov Institute of Organoelement Compounds of Russian Academy of Sciences, Vavilova St. 28, 119991 Moscow, Russia

[2] CIBER de Bioingeniería, Biomateriales y Nanomedicina (CIBER-BBN), Spain

[3] Department of Molecular Nanoscience and Organic Materials, Institute of Materials Science of Barcelona (ICMAB-CSIC), Campus UAB, 08193 Barcelona, Spain

[4] Dynamic Biomimetics for Cancer Immunotherapy, Max Planck Partner Group, ICMAB-CSIC, Campus UAB, 08193 Barcelona, Spain

[5] SPM Service, ICMAB-CSIC, Campus UAB, 08193 Barcelona, Spain

[6] Department of Molecular Biophysics and Polymer Physics, St. Petersburg State University, Ulyanovskaya St. 1, 198504 St. Petersburg, Russia

1. Partial Specific Volume

The density measurements were carried out in the density meter DMA 5000M (Anton Paar, Graz, Austria) according to the procedure of Kratky et al.[1]

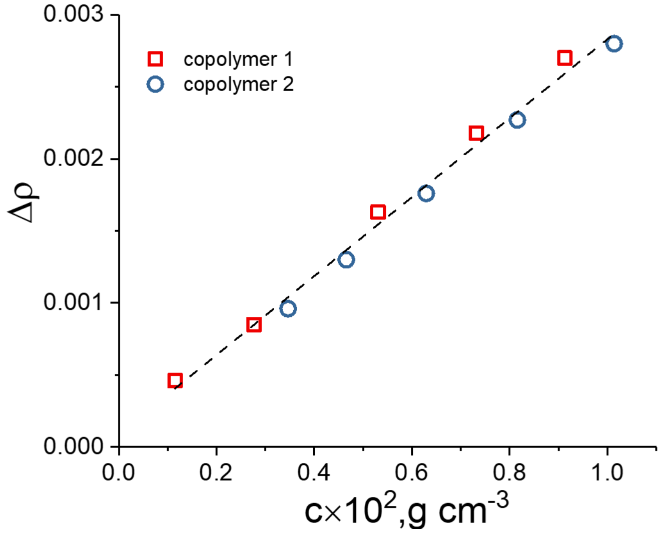

Figure S1. Partial specific volume determination for copolymers **1** and **2** in water 0.2 M NaCl solutions, T = 25°C.

2. Dissociation constants (pKa) of functional units

Table S1. Dissociation constants (pKa) of functional units.

Name	Functional (side) group	pKa exp.	References
Arginine	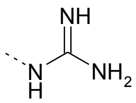	12.5	[2]
Aspartic acid	-COOH	3.9	[2]
Cysteine	-SH	8.3	[2]
Glutamic acid	-COOH	4.2±0.9	[2]
Histidine	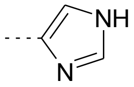	6.6±1.0	[2]
Lysine	$-NH_2$	10.5±1.1	[2]
Tyrosine	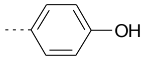	10.3±1.2	[2]
Propionic acid	-COOH	4.8±0.1	[3]
4-Methylpyridine	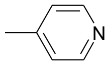	6.0	[4]
Terminal carboxy group in the peptide	-COOH	3.3±0.8	[5]
Terminal amino group in the peptide	$-NH_2$	7.7±0.5	[5]

3. Piezoresistive support

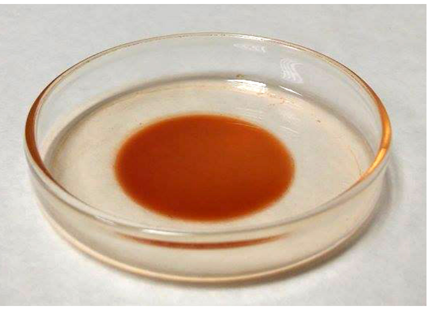

Figure S2. Picture of a flexible highly piezo resistive bilayer film-based support consisting of polycarbonate/*00l* oriented polycrystalline layer of α-(BEDT-TTF)$_2$I$_3$.[6] The conducting layer is self-assembled at the central part of the film.

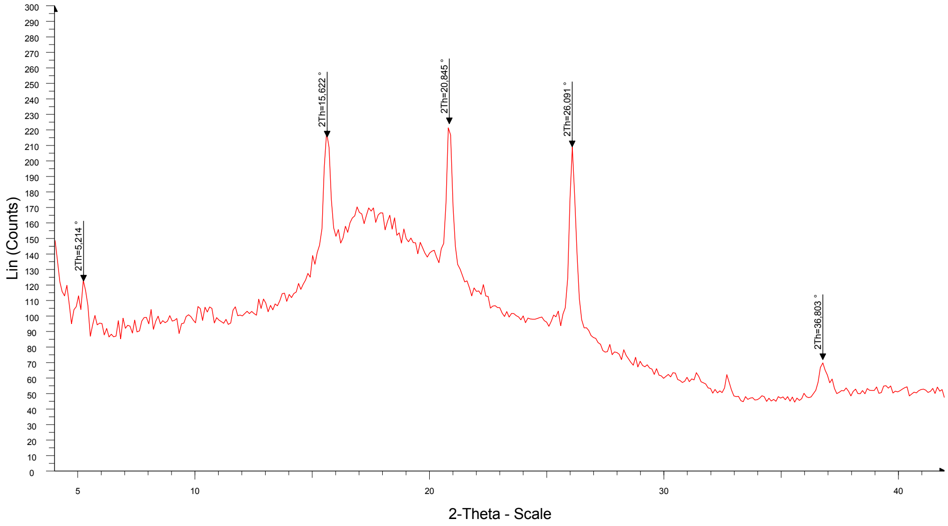

Figure S3. The X-ray diffraction pattern demonstrating the typical set of reflections for *001* oriented α-(BEDT-TTF)$_2$I$_3$ crystallites.[6]

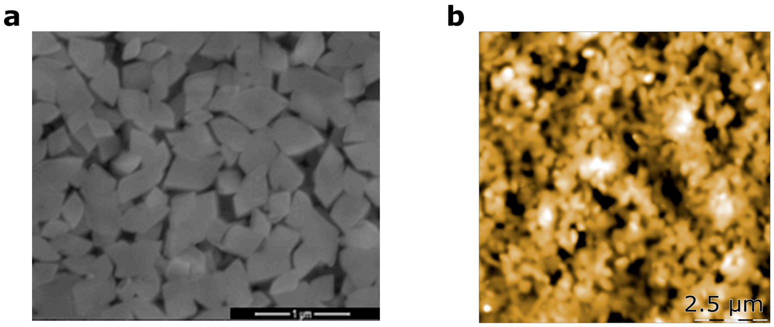

Figure S4. a) SEM image and b) AFM topography image of the conductive layer of the flexible highly piezo resistive bilayer film.

4. Analytical Ultracentrifugation

Sedimentation velocity experiments were performed using a ProteomeLab XLI Protein Characterization System analytical ultracentrifuge (Beckman Coulter, Brea, CA) equipped with conventional double-sector Epon centerpieces with an optical path length of 12 mm and a four-hole rotor (AN-60Ti). The rotor speed was 42000 rpm. Cells were filled with 420 µL of a sample solution and 440 µL of solvent (0.2 M NaCl in water). Before a run, the rotor was equilibrated for approximately 1 h at 25℃ in the centrifuge. Sedimentation profiles were obtained at the same temperature using interference optics. For the analysis of the sedimentation velocity data, the $c(s)$ model with a Tikhonov–Phillips regularization procedure implemented into the Sedfit program was used.[7] The $c(s)$ analysis involved the numerical solution of the Lamm equation assuming the same frictional ratio (f/f_{sph}) values for each sedimenting species.

Evaluated velocity sedimentation coefficients and frictional ratios were extrapolated to zero concentration using corresponding linear approximations:

$$s^{-1} = s_0^{-1}(1 + k_s c) \quad (1)$$

$$f/f_{sph} = \left(f/f_{sph}\right)_0 (1 + k_f c) \quad (2)$$

where s_0 and $(f/f_{sph})_0$ are extrapolated values of the sedimentation coefficient and frictional ratios correspondingly, k_s is the concentration sedimentation parameter (Gralen coefficient), and k_f is the concentration frictional ratio parameter. The corresponding concentration dependences of the sedimentation coefficients and frictional ratios are presented in Figures S5 and S6, respectively.

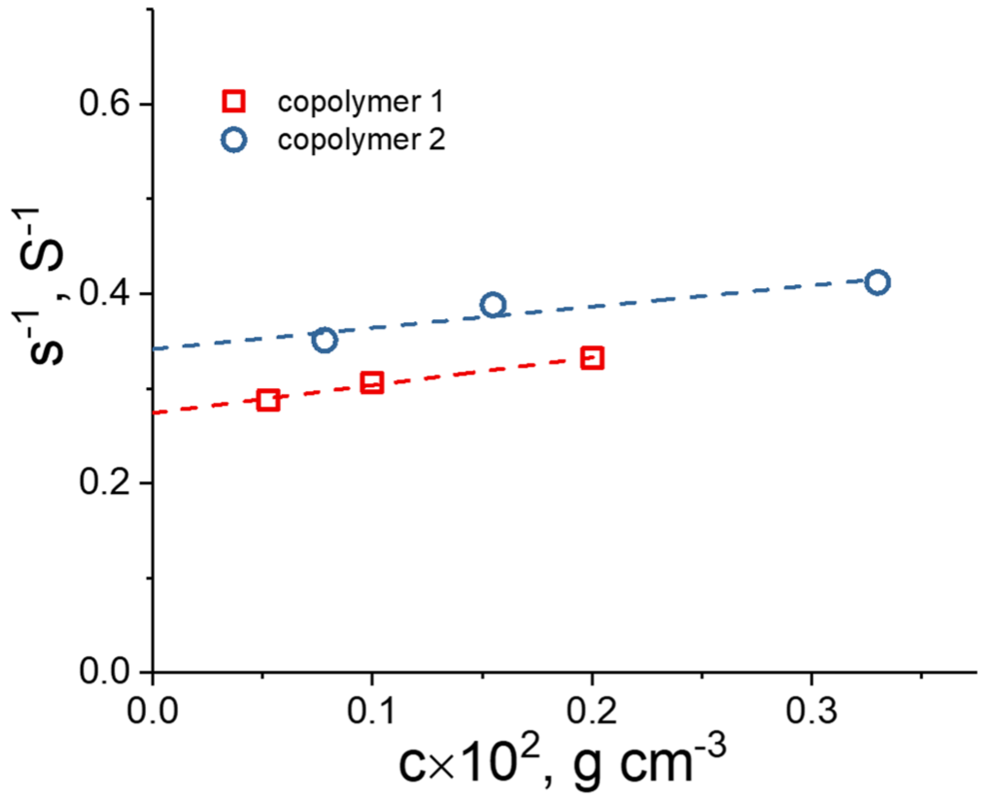

Figure S5. Concentration dependence of the reciprocal sedimentation coefficients for copolymers **1** and **2** in water 0.2 M NaCl solutions, T = 25°C.

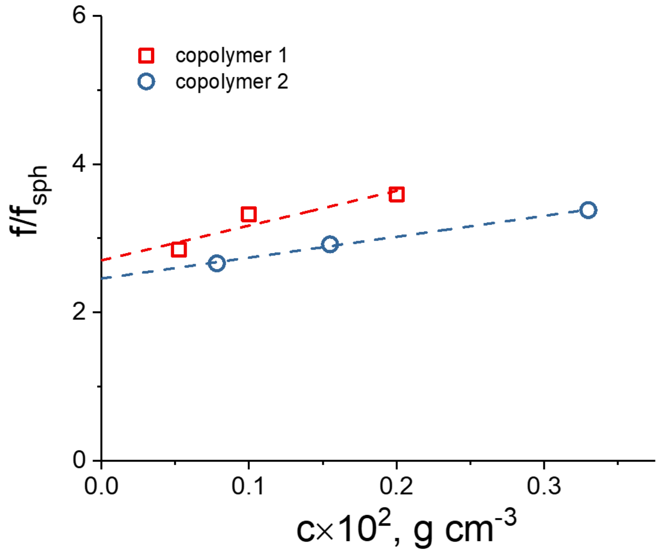

Figure S6. Concentration dependence of the frictional ratios for copolymers **1** and **2** in water 0.2 M NaCl solutions, T = 25°C.

5. AFM images of thin films of copolymer 1

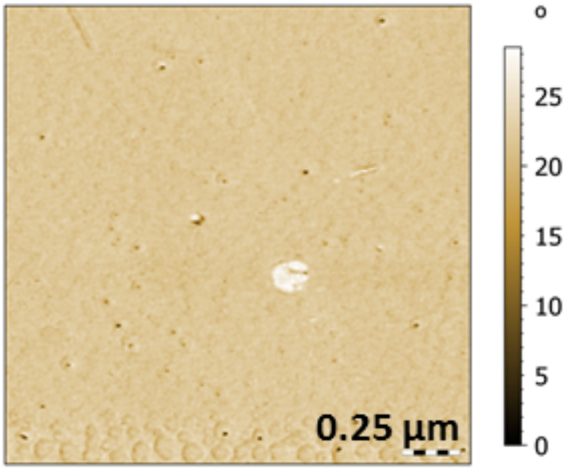

Figure S7. Bimodal phase image of the nanocavities of the self-assembled film of copolymer **1**.

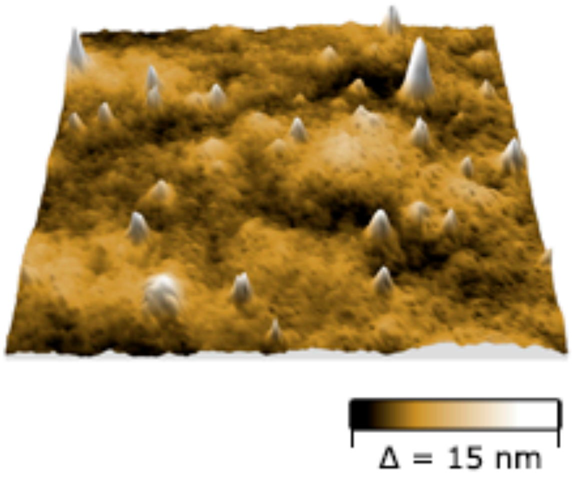

Figure S8. 3D representation of the AFM topography of myoglobin-filled nanocavities of the self-assembled film of copolymer **1**.

6. "PS-LDPE-12M" dummy sample

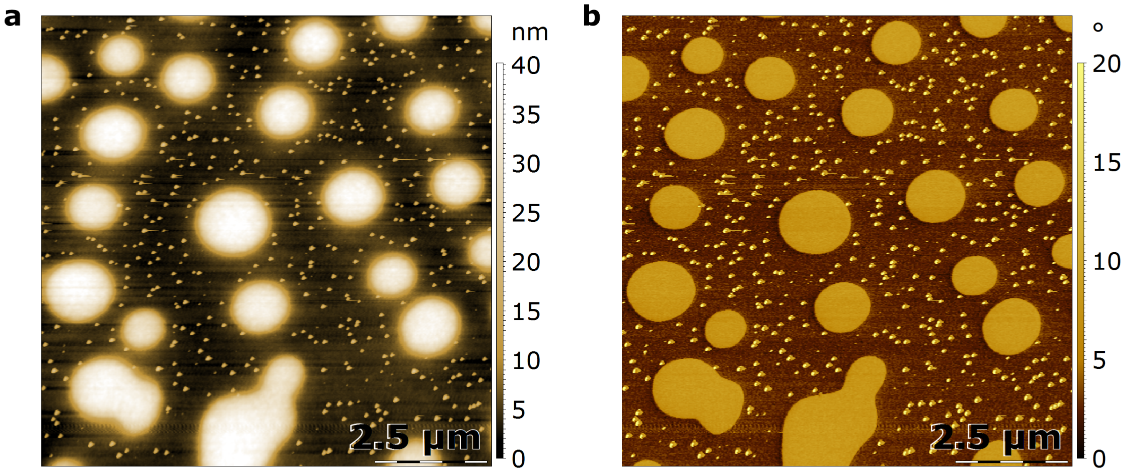

Figure S9. a) Topography and b) bimodal phase AFM images of the blend sample "PS-LDPE-12M" consisting of polystyrene with an elastic modulus of 2 GPa (bright areas) and polyolefin elastomer with an elastic modulus of 0.1 GPa (dark areas).

7. Scheme of myoglobin trapping

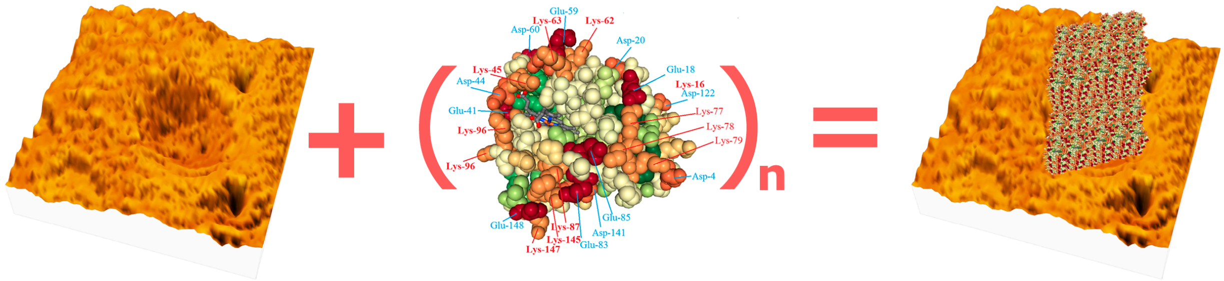

Figure S10. Scheme of myoglobin trapping by one cavity, where an "n" number of myoglobin molecules form protrusions consisting of crystalline-like myoglobin-based aggregates. In the figure, the "blue" and "red" colors of myoglobin represent negatively and positively charged groups, respectively.

8. AFM topography images of negatively and slightly positively charged biomolecules on thin films of copolymer 2

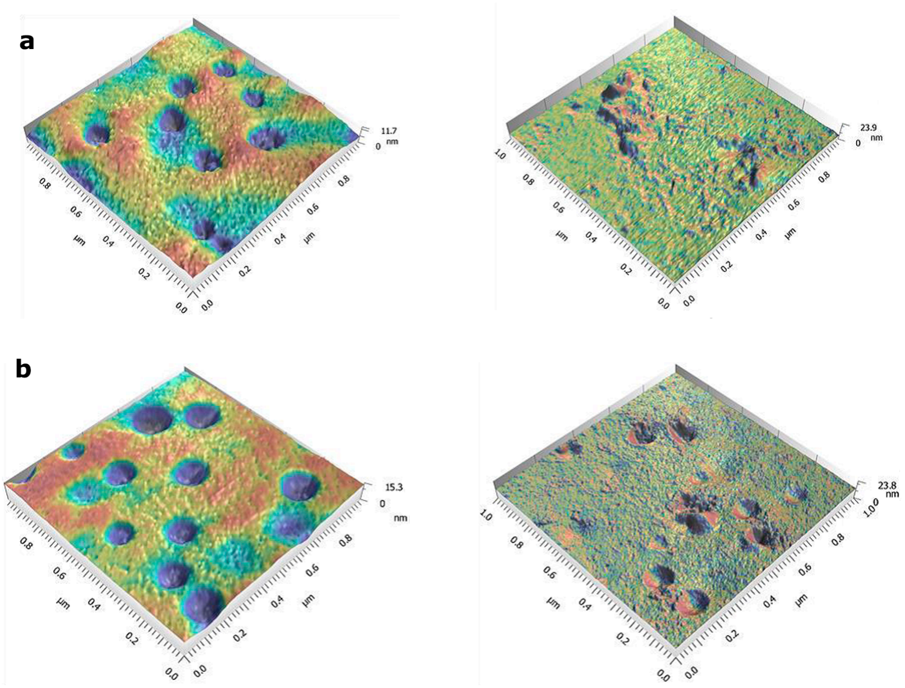

Figure S11. AFM topography images showing the surface change of a thin film of copolymer **2** after adding a) a negatively charged circular DNA (pBR322) solution and b) a water solution of myoglobin (pH ≈ 7).

9. References

(1) Kratky, O.; Leopold, H.; Stabinger, H. *Methods Enzymol.* **1973**, *27*, 98-110.

(2) Haschemeyer, R.H.; Haschemeyer, A.E.V. *Proteins. A Guide to Study by Physical and Chemical Methods*. John Wiley & Sons Inc.: Michigan (USA), **1973**.

(3) Dalai, B.; Dash, S.; Singh, S.K. *Indian J. Pure Appl. Phys.* **2014**, *52*, 24-29.

(4) Thurlkill, R.L.; Grimsley, G.R.; Scholtz, J.M.; Pace, C.N. *Protein Sci.* **2006**, *15*, 1214-1218.

(5) Grimsley, G.R.; Scholtz, J.M.; Pace, C.N. *Protein Sci.* **2009**, *18*, 247-251.

(6) Lebedev, V.; Laukhina, E.; Laukhin, V.; Rovira, C.; Veciana, J. *Eur. J. Inorg. Chem.* **2014**, 3927-3932.

(7) Schuck, P. *Biophys. J.* **2000**, *78*, 1606-1619.
